# Physician migration at its roots: a study on the emigration preferences and plans among medical students in Romania

**DOI:** 10.1186/s12960-017-0181-8

**Published:** 2017-01-19

**Authors:** Şoimita Mihaela Suciu, Codruta Alina Popescu, Mugur Daniel Ciumageanu, Anca Dana Buzoianu

**Affiliations:** 1 Department of Physiology, “Iuliu Hatieganu” University of Medicine and Pharmacy, Victor Babes Nr 8 Street, 400012 Cluj-Napoca, Romania; 2 Department of Abilities-Human Sciences, “Iuliu Hatieganu” University of Medicine and Pharmacy, Victor Babes Nr 8 Street, 400012 Cluj-Napoca, Romania; 3 Faculty of Psychology and Sociology, West University, Vasile Parvan Nr 8 Street, 00223 Timisoara, Romania; 4 Department of Pharmacology, Toxicology and Clinical Pharmacology, “Iuliu Hatieganu” University of Medicine and Pharmacy, Victor Babes Nr 8 Street, 400012 Cluj-Napoca, Romania

## Abstract

**Background:**

Migration of healthcare workers is receiving increased attention worldwide. In Europe, the creation of a border-free labor market and its expansion with the EU enlargements of 2004, 2007, and 2013 endowed health professionals with the right to provide services and to relocate to another EU Member State. For the Romanian doctors, the EU-wide recognition of the medical degree obtained in Romania has created new opportunities, while inadequate working conditions and relatively low salaries pushed many of them to search for employment abroad. As there is considerable uncertainty about the magnitude of the Romanian physicians’ exodus, we performed a survey to assess the emigration intention of future Romanian doctors.

**Methods:**

The study was conducted over three consecutive years: 2013, 2014, and 2015 at the University of Medicine and Pharmacy “Iuliu Hatieganu” Cluj-Napoca, Romania. The self-administrated questionnaire included 19 questions regarding students’ emigration intentions.

**Results:**

All the 957 license-degree students participated in the study. In this study, 84.7% of subjects planned on seeking employment abroad after graduation. A large number of the students who have participated in the study have already started preparing for emigration, 21.7% of those who wished to migrate had already performed at least one Erasmus mobility in their country of choice, 44.5% have been enrolled in a language course, and 42.7% have searched for jobs on the Internet.

**Conclusions:**

The majority of Romanian medical students considering migration see it as a serious alternative to the continuation of their professional training started in Romania. The findings of this study are upsetting and can impact both policy crafting and future research. Structural reforms in the healthcare provisions are needed in order to facilitate the retention of medical personnel. Romanian policy makers need to devise a comprehensive national health workforce plan to deal with physician migration.

**Electronic supplementary material:**

The online version of this article (doi:10.1186/s12960-017-0181-8) contains supplementary material, which is available to authorized users.

## Background

The challenges of health professional mobility and migration are not new: they have been identified and observed across decades since the first reports had been published in 1978 [1]. The demand on human resource in medicine is rapidly growing worldwide for a number of demographic and epidemiological conditions [2]. Health workers are under pressure for a number of reasons: high cost of training, attrition, migration, and increasing demand in the aging population. The main migration flow of health workers is from less developed countries to more developed ones, a fact known as “brain drain” [3]. There is an established connection between an adequate level of staffing and positive healthcare outcomes [4].

The migration patterns and the extent of healthcare professionals’ migration from the developing countries to the developed countries has been a constant focus for research [5–7].

In Europe, the creation of a border-free labor market and its expansion with the EU enlargements of 2004, 2007, and 2013 endowed health professionals with the right to provide services and to relocate to another EU Member State [8].

Surveys to analyze the migration intentions of healthcare professionals after joining the EU were conducted in Poland, the Czech Republic, Hungary, Lithuania, Croatia, and Estonia [9–12].

The driving forces for the migration of healthcare professional in the EU are different. Jinks et al. [13] investigated the movement of doctors from EU countries to the UK and concluded that the main driving forces for migration to the UK were reduced employment chances in the home country or motivation to get a better training in the UK. In all the new EU member states (for example Poland, Hungary, Lituania, Estonia), the main motivation for emigrating is a better pay in the host country [12].

Romania, who is a member of EU since 2007, deals with a health workforce crisis linked to migration [14].

The public sector in Romania is undergoing dramatic changes as the state is coming to terms with the post-Communist era, with the (neo)liberalization encouraged further by the EU accession in 2007, and with the impact of austerity measures implemented as a result of IMF loans in 2010 [15]. In the past few years, the situation in the public health system has been affected by a mix of contributing factors, including limiting economic measures, a public sector salary cut of 25%, a recruitment freeze, and budget constraints on hospitals’ staffing. In this context, Romania may primarily be thought as the “sender” country of migrant health workers to Western Europe. The brain drain of the Romanian elite is nowadays considered a “fourth wave” of migration as of 1989, with the medical profession being one of the most prominent occupations mentioned in migration studies [16].

Europe’s aging population, the free movement of labor, and mutual recognition of qualifications are creating new opportunities for Romanian medical doctors, while inadequate working conditions and relatively low salaries push many high-skilled healthcare workers to search for employment abroad. As the living standard in the Western EU member states exceeds the living standard of the new ones, economic incentives to move abroad are high, thus contributing to inequalities in the healthcare provision between them [7]. For example, a resident in Romania earns around 200 Euros, whereas residents in other EU countries can earn much more. [14].

Obtaining accurate data on migration of healthcare workers is highly problematic in Romania [14]. Official data is limited to the number of certificates of conformity for practicing medicine in the EU (although these requests do not automatically imply the migration of the health professionals). The main indicator is the “intention to leave” collected by the Romanian College of Physicians [14, 17]. Intention-to-leave data from Romania seems to indicate continuing high outflows of medical doctors, more than 300 certificates per month were issued to Romanian medical doctors in 2010 [18]. In 2014, 2450 certificates of conformity were issued. Therefore, this number can serve as an estimate for the number of doctors who left Romania for work abroad in 2014.

A further complication is the legal right in the public sector to solicit up to 2 years of unpaid leave of absence with no obligation to disclose purpose or destination. This enables staff to try out the options of working abroad, to take short overseas contracts, and ultimately to resign if they find a successful long term job abroad [19].

Another indirect indicator is the total number of registered doctors in Romania. This number was 39 000 in 2014. The total number of doctors in Romanian hospitals has steadily decreased from 20 648 doctors in 2011 to 14 487 in 2012 and to 13 521 doctors in 2014 [19]. The Romanian College of Physicians reported that “between 2007 and 2013, 14,000 medical doctors left their jobs in the national public health system and chose to practice abroad” [14].

A considerable public concern has been raised in Romania after the 2007 EU accession. An important emigration wave was predicted, especially among highly educated young professionals [16]. In an analysis of mass-media statements, Toader [20] indicates the media awareness of a large number of healthcare workers who left for other EU host countries since Romania’s accession. Among the headlines quoted, we can mention statements like: “4,000 doctors left the country in the past 2 years and almost 5,000 others are also ready to leave it” [21] and “The doctors’ exodus will determine the collapse of the healthcare system in less than 10 years” [22].

Similar predictions about the potential disaster of physician migration were formulated for other new EU member states after the accession [23, 24]. However, they turned out to be overestimations, since the annual outflows from the new member countries rarely exceeded 3% of the domestic healthcare workforce [18].

Hardy et al. [15], based on previously published figures [25, 26]), estimate that 3% of doctors and 5% of nurses leave the country each year. However, this number may be underestimated, as 20 to 40% of the total health worker population expresses a desire to work abroad.

## Methods

### Aim of the study

As there is considerable uncertainty about the magnitude of the Romanian physicians’ exodus, we performed a survey to assess the emigration intention of future Romanian doctors. The study was conducted over three consecutive years: 2013, 2014, and 2015.

### Sample

The subjects of this study were 957 Romanian citizens, graduates of the faculty of medicine, who studied medicine in the Romanian language at the University of Medicine and Pharmacy “Iuliu Hatieganu”.

Inclusion criteria: medical school graduates who are Romanian citizens and who passed the theoretical and practical test of the license exam.

Exclusion criteria: medical school graduates who are not Romanian citizens and medical school students who are Romanian citizen but failed on the theoretical or practical tests of the license exam.

#### Procedure

The study was conducted in close collaboration with the student organization, we held several sessions with the leaders of the students in order to communicate information about the purpose of the study. During the university years, one third of the students also participated in counseling sessions on specialty choice and they were also notified about this study.

The questionnaire was distributed to students during the licensing exam, before the public defense of theirs dissertations. The students were invited to participate after the survey’s purpose and administration issues were explained. Participation in the survey was voluntary. Students did not receive any incentives to participate in the study. For the students, the direct benefit of participation in this research was the opportunity to express an opinion about a subject that is of interest to them: where they will be practicing medicine after residency and to contribute to the collection of data that could shape the public policy regarding health workforce in Romania.

The study received the approval of the Research Ethics Committee of our University. Response rate was 100%.

#### Questionnaire

The self-administrated questionnaire (Additional file 1) used for the study includes 21 questions regarding students’ emigration intention. The questions were centered on willingness to go work abroad and the possible reasons for such a decision. Participants were asked to specify how advanced their plan to work abroad was, when and for how long they planned to go. Several other questions on push and pull factors for migrations, the target country, and job preference were asked. In addition, demographic data about the students were collected (see Additional file 1).

#### Statistical analysis

Descriptive statistical analysis was performed to calculate means, standard deviations, and frequency. The distribution of the studied variables was examined using Shapiro-Wilk’s and Kolmogorov-Smirnov’s tests. Statistical significance was assumed at *p* < 0.05. The Mann-Whitney *U* test and Kruskal-Wallis test was used to compare groups with non-normal distributions. Chi-square was used to compare frequencies. The analysis was performed in SPSS version 20.

## Results

### Characteristics of the population

The study included 957 students, females made up 71.5% of the sample; 824 students (86.1%) came from urban environments (Table 1). Regarding relationship status, 58.2% of the students were single, 31.8% in a relationship, and 10% were married. The majority of students’ parents (62.3% of mothers and 65.3% of fathers) had a university degree, 19.9% of students’ parents were doctors. The student’s age ranged from 24 to 34 years, with the mean age 25.03 (±1.01) years. The students assessment of their household economic situation was 2.6% living in precarious conditions, 23.5% that cannot afford everything needed for a normal life, 66.7% that can afford everything needed for a normal life, and 7.2% that can consume without any restrictions. 31.2% of the participants desired to work in a hospital setting, 1.8% in a public outpatient practice, 10.1% aspired to work in a private practice, while 56.8% wanted to work in both a hospital setting and a private practice.

**Table 1:** Characteristics of the population

	2013		2014		2015		Total	
	*N*	%	*N*	%	*N*	%	*N*	%
Gender								
Male	89	27.8	87	27.0	97	30.8	273	28.5
Female	231	72.2	235	73.0	218	69.2	684	71.5
Residence								
Urban	268	83.8	278	86.3	278	88.3	824	86.1
Rural	52	16.2	44	13.7	11.7	37	13.9	133
Relationship status								
Single	174	54.4	197	61.2	186	59.0	557	58.2
In a relationship	110	34.4	94	29.2	100	31.7	304	31.8
Married	36	11.2	31	9.6	29	9.2	96	10.0
Economic situation of the household								
Living in precarious conditions	10	3.1	10	3.1	5	1.6	25	2.6
Can manage generally	90	28.1	72	22.4	63	20.0	225	23.5
Can afford everything needed for a normal life	202	63.1	225	69.9	211	67.0	638	66.7
Can consume without restriction	18	5.6	15	4.7	36	11.4	69	7.2

### Desire to emigrate

Table 2 shows the distribution of intention and willingness to work abroad. 84.8% of the respondents have more or less developed a plan to seek employment abroad after graduation. Respondents were asked to estimate the probability of migrating on a 0–100 scale (ranging from 0—I don’t want to leave, 25—unlikely to leave, 50—likely to leave, to 100—definite leaving intentions) (Table 3). The average probability to emigrate was 42.17% (±30.24), with no significant difference between males and females (Man-Whitney *U* = 39 690.00, *p* = 0.510). 15.2% of the students estimate a 0 probability to leave, 34.2% a 25 probability score, 35.4% a 50 probability score, and 15.2% estimate the probability to emigrate at 100.

**Table 2:** Distribution of medical students planning to work abroad

	2013		2014		2015		Total	
	*N*	%	*N*	%	*N*	%	*N*	%
Do not want to go	48	15	49	15.2	49	15.6	146	15.3
Vague plan	99	30.9	110	34.2	99	31.4	308	32.2
Developed plan	121	37.8	114	35.4	129	41.1	364	38
Definite plan	52	16.2	49	15.2	38	12.1	139	14.5
Total	320	100	322	100.0	315	100	957	100

**Table 3:** Timeframe for migration

	2013		2014		2015		Total	
	*N*	%	*N*	%	*N*	%	*N*	%
I don’t plan to leave	48	15	49	15.2	49	15.6	146	15.3
I will leave as soon as I graduated	38	11.9	33	10.2	19	6	90	9.4
In the first year of residency training	52	16.2	108	33.6	43	13.7	203	21.2
In the 2–3 year of residency training	136	42.5	65	20.2	130	41.3	331	34.6
After specialization	21	6.6	33	10.2	38	12.1	92	9.6
After I work a few years in Romania	25	7.8	34	10.6	36	11.4	95	9.9
Total	320	100	322	100.0	315	100	957	100

The percentages of students who want to leave Romania stay rather constant across the years (around 15% for the students who do not want to go, around 30% for those with a vague plan, around 35–40% for those with a developed plan, and around 15% for those with a definite plan). This relatively stable distribution can suggest that there are no major changes to be expected. The 3% dip in de definite plan category in 2015 is something to be monitored in the coming years. Based on the data available so far, the dip in 2015 could be attributed to a cohort effect.

### Timeframe for migration, preferred length of stay abroad, and host country

21.2% of the students plan to leave 1 year after graduation, 34.6% intend to leave within 2 to 3 years after graduation, and 9.4% do not even foresee to pass a residency training admission exam in Romania. Only 15.3% of the students do not have any plans for migration (see Table 2).

Most students want to go abroad for a short period—35.2% plan to stay abroad a few months, 26.1% intent to stay abroad for several years and then come back to practice in Romania, and 23.4% of the students have a permanent emigration plan (see Table 4). Among the students who are planning to emigrate the preferred host countries are Germany (34.1%), France (20.0%), and Great Britain (19.6%). 28.5% of the student do not know yet to which country they want to emigrate.

**Table 4:** Preferred length of stay abroad

	2013		2014		2015		Total	
	*N*	%	*N*	%	*N*	%	*N*	%
I don’t plan to leave	48	15	49	15.2	49	15.6	146	15.3
For several months	108	33.8	109	33.9	120	38.1	337	35.2
For several years	83	25.9	93	28.9	74	25.3	250	26.1
Permanently	81	25.3	71	22	72	22.9	224	23.4
Total	320	100	322	100.0	315	100	957	100

### Concrete departure preparation

The students who plan to emigrate have taken concrete steps towards emigration goals, including spending time abroad in Erasmus student mobility (21.7%), enrolling in language courses (44.5%), searching jobs on the Internet (42.7%), participating in job fairs for healthcare professionals (40.1%), and contacting Romanian physicians who are working abroad (75.2%). There are significant differences in the level of preparation between the students who plan to emigrate and the students who do not want to leave Romania (see Table 5).

**Table 5:** Cross tabulation of concrete departure preparation and migration intention

Concrete departure preparation			Migration intention		*χ* ^2^	*p*
			Do not emigrate	Emigrate		
Taking part in Erasmus student mobility	No	*N*	138	635	20.96	0.000
		%	94.5	78.3		
	Yes	*N*	8	176		
		%	5.5	21.7		
Enrolling in language courses	No	*N*	110	450	20.09	000
		%	75.3	55.5		
	Yes	*N*	36	361		
		%	24.7	44.5		
Job search on the Internet	No	*N*	115	465	23.8	0.000
		%	78.8	57.3		
	Yes	*N*	31	346		
		%	21.2	42.7		
Jobs fairs for healthcare professionals	No	N	117	486	21.64	0.000
		%	80.1	59.9		
	Yes	*N*	29	325		
		%	19.9	40.1		
Talking with Romanian physicians who are working abroad	No	*N*	59	201	15.26	0.000
		%	40.4	24.8		
	Yes	*N*	87	610		
		%	59.6	75.2		

### The factors influencing the migration decision

In this section, we are giving an account of the main factors influencing the decision to work abroad. These factors could be the potential subjects of inquiry for health policy developers if the general aim is the reduction of the prospective migration flows of Romanian healthcare professionals. Respondents were asked to rank the following reasons for resettlement on a 0–100 scale: higher wage abroad; better living and working condition abroad; I am disappointed with the Romania healthcare system; to gain living and working experience abroad; personal reason (my partner want to work/is working abroad); more professional opportunities; shortage of residency opportunities in my chosen specialty in Romania; and lack of job vacancies in my chosen specialty in Romania (see Table 6).

**Table 6:** Reasons for migration

	Do not emigrate		Emigrate		Man-Whitney *U p*
	Mean	Std. deviation	Mean	Std. deviation	
Higher wage abroad	61.62	±37.94	82.57	±22.52	0.000
Better working and living condition abroad	65.84	±37.28	88.91	±34.58	0.000
I am disappointed in the healthcare system in Romania	68.24	±34.35	77.44	±34.47	0.000
To gain living and working experience abroad	31.5	±31.42	59.61	±30.95	0.000
Relationship status	12.94	±24.12	25.17	±33.51	0.000
More professional opportunities	46.27	±35.16	73.08	±27.02	0.000
Shortage of residency places in my chosen specialty	21.37	±31.51	35.85	±35.72	0.000
Lack of working places in my chosen specialty	26.41	±33.89	54.35	±37.88	0.000
Total score	3344.22	±239.33	472.17	±158.86	0.000

As a main pull factor for migration (see Table 6), students who want to emigrate highly rank the elevated wages and better living and working conditions abroad (88.91 ± 34.58 and 82.57 ± 22.52). Similarly, they rank their discontent with the Romanian healthcare system (77.44 ± 34.47). Another important factor is the lack of working places in Romania (54.35 ± 37.88). The least frequently reported migration factor is relationship status (my partner wants to work/is working abroad), with an average of 25.17 ± 33.51. We find significant differences between the students who want to emigrate and the ones who do not want to emigrate.

The majority of students are not satisfied with the anticipated salaries they could earn working in the Romanian (implicitly) public health system (see Table 7).

**Table 7:** Distribution of satisfaction with anticipated salaries in the Romanian health system (%)

	*N*	%
Not satisfied at all	341	35.6
Rather not satisfied	470	49.1
Quite satisfied	59	6.2
Very satisfied	5	0.5
Difficult to say	82	8.6
Total	957	100.0

The Spearman’s rho correlation coefficient between the concrete departure preparation (the variable is a sum of all “yes” answers to possible actions, like Erasmus mobility, language courses, job search, contacts with Romanian physicians working abroad) and reason for migration was 0.314, with *p* < 0.01.

The students who are determined to migrate score higher on reasons for migration total score and on concrete departure plans (see Table 8).

**Table 8:** Relationship between the departure plans, professional dissatisfaction and migration intention

		Reasons for migration—total score	Concrete departure plans—total score
Do not want to go	Mean	334.22	1.36
	*N*	146	146
	Std. deviation	239.33	1.02
Vague plan	Mean	474.87	2.55
	*N*	308	308
	Std. deviation	126.712	1.38
Developed plan	Mean	505.10	4.26
	*N*	364	364
	Std. deviation	111.53	1.44
Definite plan	Mean	524.79	4.74
	*N*	139	139
	Std. deviation	142.89	1.47
Kruskal-Wallis *W*		119.81	399.69
*p*		0.000	0.000

## Discussion

This is the first study of its kind in Romania, aiming to provide an in-depth analysis of prospective physician migration, with a particular emphasis on the attitudes and practices undertaken by medical graduates in relation to choosing a career abroad.

In this study, 84.7% of the respondents are considering to seek employment abroad after graduation. The total percentage of students that are considering emigration (84.7%) is higher than in other Eastern European countries. For comparison, Polish students estimated the likelihood of emigration to be around 50% [11]. 60% of medical residents from Lithuania stated that they intended to emigrate, 15% of them permanently [27], and only 45% of the Czech physicians are contemplating emigration [28]. One third of the final year medical students from Croatia reported their willingness to permanently leave the country in search of employment elsewhere [10]. In 2004, surveys conducted in Central and Eastern Europe found that 10.4% of Polish, 15.6% of Czech, and as many as 24.7% of Hungarian physicians contemplated migration [12]. Interestingly, these forecasts often seem to overestimate the true rate of emigration, as joining the EU was rarely associated with a percentage of emigrated physicians higher than 3% [18].

Regarding the Romanian sample, female alumni made up the majority of our sample. This is due to the fact that a higher number of female students are enrolled for medical studies. The top destination choice among those who wish to emigrate is Germany (with 34.1%). Among reasons for pursuing a career abroad, the mean score for “higher wage than in Romania” was most notable. There were no gender or marital status differences in correlation with the importance of a better payment abroad. In Romania, public health sector wages represent, on average, around 15% of the levels typically paid in the old EU member states, and even if differences in the living cost are taken into account, migration provides an attractive financial gain [15].

Other reasons for migration included disagreement with the Romanian healthcare and residency system, as well as better prospective quality of life abroad. In the past few years, the tradition of Romanian informal, “under-the-table” payments offered by patients to doctors and nurses has been highlighted in the media in a manner that vilifies the medical professions and this bad press is seen as a further possible reason for dissatisfactions and consequently for the migration of young doctors [15].

In our study, practice preferences upon graduation indicate that about 33% of the medical alumni desire to practice exclusively in the public sector rather than the private sector (10.1%). Another 56.8% intend to work simultaneously in a public hospital setting and private practice. These preferences are expressed in the context of a constant decline of number of doctors practicing in Romanian public hospitals. The public hospitals present the number of doctors decreasing from 20 648 in 2011, to 14 487 in 2012, and to 13 521 doctors in 2014 [19]. The main reason for health professionals to stay in the public sector may be for future training, academic tenure, and gaining experience before joining the private sector.

Loss at the societal level, caused by physicians’ migration for work, must be understood in the wider context of the loss of an educated professional resource for which the costs for qualification are very high. The state invests in the education of a physician for a longer period of time and the costs are much higher than for other professions. Not to mention the fact that doctors and nurses are not easily replaceable. The direct, average, costs for the training of a physician are around 6660 Euros for undergraduate education and 4660 Euros for specialty training [29]. Theoretically, such a financial burden in a public educational system brings a serious issue of concern regarding the role of the state to damper the migration flow of health professionals.

At the public policy levels, the economic crisis and the lack of efficiency of the Romanian Administration have generated negative effects on the Romanian society. The massive white gown migration is a phenomenon new to Romania, and this type of migration is very different from the migration of unskilled workers. The long-term effects of this migration are not known yet, but might affect many people, especially the communities in small towns and rural areas, where poverty rates can be twice as high as in urban areas and where they do not have immediate access to a full time medical assistant/nurse or a doctor [14].

Physicians’ migration for work can also turn into permanent migration much easier than previous waves of Romanian migration: as the doctors have better wages than unqualified workers and it is much easier for them to bring their family along. Therefore, losing 1000 doctors per years is a lot when comparing this number to the total number of doctors in Romania (39 000 in total and 13 521 working in the hospitals in 2014) [19]. This number raises serious questions regarding a possible national vulnerability caused by the emigration of Romanian health workers to the developed countries in Western Europe.

There are three main arguments used by physicians in favor of migration: low salary, the lack of social status and the continuous deterioration of the public perception, constant lack of government interest for investment, and appropriate public policies in the healthcare sector. A large number of the students who have participated in the study have already started preparing for emigration, 21.7% of those who wished to migrate had already performed at least one Erasmus mobility in their country of choice, 44.5% have been enrolled in a language course, and 42.7% have searched for jobs on the Internet.

The reasons for potential emigration among graduating medical students in Romania were similar to those reported in other European countries. For instance, major reasons for leaving Lithuania were higher salary, better professional opportunities, and better quality of life [27], similarly to the findings among Czech physicians [28] and students from Poland [11].

From an EU labor market perspective, free mobility of doctors can be a way to balance supply and demand for health workforce. Underemployment has led Romanian health professionals to seek work elsewhere in the EU. For destination countries, free mobility can contribute to health system performance when foreign health professionals fill services gaps [30]. In France, in 2007, 40% of newly registered anesthetists and 20% of newly registered pediatricians were EU nationals, mainly from Romania [31].

Returning health professionals may increase expertise in the home system when they improve their skills and qualifications abroad [32]. From this perspective, 26.1% of the students who declare they want to stay abroad for several years and then come back to practice in Romania may prove to be an asset.

Mobility can also provide a policy stimulus to tackle workforce issues. In 2010, some 3800 publicly employed Czech doctors joined the protest movement “Thank you, we’re leaving,” threatening to collectively resign and subsequently obtained salary increases and improvements to the educational system [30, 33].

We didn’t find any statistics regarding migration of physicians, how many, where they go, what age or professional experience, and where they graduated. Romania should further strengthen the availability of health workforce data, which should cover migration issues. This would facilitate policy and decision-making in Romania. Despite a large number of medical professionals having already migrated, it is unclear whether any of them returned, from where, and where they are currently employed (public or private health sector). A centralized database for health workforce data that tracks health worker mobility would allow for better public policy decisions [14].

Migration is an expression of liberty and individual choice. However, the state invests in the training of medical personnel and will lose this investment if the medical students will migrate after graduation. As a policy, the government could choose to try to penalize migration or to stimulate the retention of medical personnel in the country.

The Government could propose a tax on the migration of highly skilled medical personnel (the doctors will have to work in the country a certain number of year after graduation or reimburse the cost of education). But if this tax is proposed only for medical personnel, this will create discrimination and will be rejected as non-constitutional. Additionally, such a penalty goes against the EU principle of free circulation of workforce and free movement and therefore would not stand a chance in the EU Court of Justice. An alternative option would be to develop some bilateral agreements between Romania and the destination countries to arrange for some financial compensation. Open methods of coordination currently existent within the EU would provide different ways to arrange for such an option.

So far, confronted with situation created by the migration of doctors, the first measure of the Government was to raise the medical personal remuneration by 25% (Government Emergency Ordinance nr. 35/2015) starting with 1 October 2015. The effects of this measure will satisfy only the financial problems of the doctors. Still, the Government will need to invest in the equipment of the hospitals, training of physicians, and in abolishing corruptions in the medical system [34].

Structural reforms in the healthcare provisions are needed in order to facilitate the retention of medical personnel. The institution of contracts to retain the new graduates for 5 years can only lead to building resentment on behalf of the medical personnel affected by this measure and to costly and time-consuming juridical battles. The results of this research suggest that the primary concern remains related to personal finances. Therefore, the measures already taken to increase the salaries of medical personnel can be included as a first step towards the desired direction.

Next to addressing individual financial security, additional measures need to be taken in order to develop a healthcare system that provides attractive career options for the personnel (current and new graduates). Structural financial investments need to include financial support regarding professional integration of young professionals, coupled with facilitating the re-building access to medical care provision in small to medium size cities, and rural areas would provide attractive work options. Extensive measures to combat vilification of the medical profession in the media could help motivate young professional towards building their career in Romania. Additional measures could also include the support for professional integration of physicians and nurses who are working abroad back into the Romanian healthcare system.

### Strengths and limitations

This study has a number of strengths. Firstly, the perfect response rate (100%) decreases the likelihood of a response bias. Secondly, this is the first study describing the demographics of a large sample of graduates of a Romanian medical faculty.

The most important limitation to consider for our study is that it was restricted to just one main Romanian faculty of medicine. Although the studied alumni population is rather heterogeneous, with individuals coming from all regions of the country, it cannot predict the overall situation in Romania. This implies that further studies should be conducted on a larger scale, with other medical schools participating in the same survey, in order to minimize bias as well as to ensure a broader view of the current situation. Also, studies can be done on residents, who choose to migrate, to see the personal and professional factors that influence the decision to turn a temporary mobility for specialization in a permanent migration.

Assessing intentions, and not actual behavior, represents another limitation of this study. We were not able to identify in literature any study linking migration intention to actual migration behavior. However, even if we were to assume a less than strong association, the percentage of those intending to emigrate abroad is alarming in the general context of the actual migration of Romanian physicians.

Another limitation of the study is the deployment of quantitative data and the lack of nuance brought by qualitative data. In-depth ethnographic research could lead to a better understanding of personal drives of individuals as well as to a better understanding of the migration in the context of a globalized pressure on the health workforce to comply to new rules of the disempowerment of professionals under the pressure of the neoliberal world order.

## Conclusions

The majority of Romanian medical students considering migration see it as a serious alternative to the continuation of their professional training started in Romania. The findings of this study are disturbing in terms of the future migration of Romanian physicians and have implications for both policymaking and future research. Romanian policy architects need to devise a comprehensive national health workforce plan to deal with physician migration. This should take into account the narratives and behaviors of both stayers and leavers. Such a plan should carefully address the financial incentives in the Romanian public sector, the recruitment freeze, status issues, and other factors contributing to the migration of physicians, such as the quality of residency training. Specific policies targeting medical students should prove beneficial in the long run, as the college life is the prime time for molding attitudes of future physicians. Special allocations or status reinforcements should be devised for those who do not intend to migrate in the future. There is a need for more research investigating the impact of migration on the effectiveness and efficiency of healthcare systems in both the home and host countries.

## Additional file

Additional file 1: Questionaire regarding migration intent. (DOCX 41 kb)
