# Supplementary material for: Physician migration at its roots: a study on the emigration preferences and plans among medical students in Romania
Source: Hum Resour Health. 2017 Jan 19;15:6. doi: 10.1186/s12960-017-0181-8 (PMC5247802; doi:10.1186/s12960-017-0181-8)
Supplement: Additional file 1: — Questionaire regarding migration intent. (DOCX 41 kb) [file 12960_2017_181_MOESM1_ESM.docx]

**APPENDIX**

**Questionnaire regarding migration intent**

**Dear student, please answer the following questions.**

**1. Age _____________**

**2. Gender** 1. male 2. female

**3. Residence**

1. urban

2. rural

**4. Relationship status**

1. single

2. in a relationship

3. married

**5. Parents level of education**

**mother:**

1. high school

2. higher education

**father:**

1. high school

2. higher education

**6. Is one of your parents a medical doctor?**

1. Yes
2. No

**7. Economic situation of the household**

1. Living in very poor conditions
2. Can’t afford everything needed for a normal life
3. Can afford everything needed for a normal life
4. Can consume without any restrictions

**8. Your GPA in the 6^th^ year:** 1. *over 9.5* 2. *9-9.49* 3. *8.50-8.99* 4. *8-8.49* 5. *under 8*

**9. Where do you plan to pass the residency exam?**

In Romania

1. Yes
2. No

In a foreign country

1. Yes
2. No

**10. What specialty do you want to choose?**.............................

**11. Where do you want to work after residency?**

1. Public health system, in a hospital
2. Public health system, outpatients care
3. Private system
4. Combination between public and private practice

**12. Do you plan to seek employment abroad after graduation?**

**Please estimate the your probability of leaving Romania on a scale ranging from 0 to 100. ______________**

**Also please mark the following categories where you estimate your probability of leaving the country.**

1. 0 % means I don't want to leave
2. 25% It is unlikely that I will leave
3. 50% It is very likely I will leave
4. 100% I am certain I will leave

**13. In which country do you plan to seek work?** ...........................

**14. If you want to leave Romania, when do you plan to leave?**

1. I don’t want to leave the country
2. Immediately after graduation, I don’t pass the residency in Romania
3. In the first year if residency
4. After 2-3 year as resident
5. After I finish my residency
6. After I will practice medicine 2-3 year in Romania to see if I can manage

**15. If you plan to leave Romania, how long to you think will be abroad?**

1. I don’t leave the country
2. I’m planning to stay abroad a few years then come back in Romania
3. I’m planning to do me specialization abroad then came to practice in Romania
4. I will emigrate permanently

**16. Below are some reasons why a physician might want to emigrate. Please note notes from 0 to 100 how important for you for these reasons:**

|  | 0-100 |
| --- | --- |
| Better payment abroad |  |
| Better working condition |  |
| I am disappointed in the Romanian health system |  |
| I want to gain life experience by living and working abroad |  |
| The person I am in relationship with wants to leave the country |  |
| Better professional opportunities abroad |  |
| There are no place to do the specialty I want in Romania |  |
| I will not have a job in Romania after residency |  |

**17.** **How satisfied are you with current wage level of a resident in Romania?**

1. Absolutely not satisfied

2. Not satisfied

3. Satisfied

4. Very satisfied

5. I don’t know

**18. Have you been abroad in a medical school with Erasmus type mobility?**

1. Yes
2. No

If you have answer yes, please write the name of the country_________________

**19. Have you made preparations for your emigration:**

Enrolled in language courses Yes No what language__________

Searched job postings online? Yes No

Attended medical job fairs? Yes No

Spoken with Romanian doctors who work abroad? Yes No
